# A locus in *Pristionchus pacificus* that is responsible for the ability to give rise to fertile offspring at higher temperatures

**DOI:** 10.1242/bio.018127

**Published:** 2016-07-18

**Authors:** Mark Leaver, Simone Kienle, Maria L. Begasse, Ralf J. Sommer, Anthony A. Hyman

**Affiliations:** 1 Max Planck Institute for Cell Biology and Genetics, Pfotenhauerstrasse 108, Dresden 01307, Germany; 2 Max Planck Institute for Developmental Biology, Department of Evolutionary Biology, Spemannstraße 37, Tübingen 72076, Germany

**Keywords:** Temperature, *Pristionchus pacificus*, Fertility, Mapping, Natural isolates

## Abstract

Temperature is a stress factor that varies temporally and spatially, and can affect the fitness of cold-blooded organisms, leading to a loss of reproductive output; however, little is understood about the genetics behind the long-term response of organisms to temperature. Here, we approach this problem in the model nematode *Pristionchus pacificus* by utilising a large collection of natural isolates with diverse phenotypes. From this collection we identify two strains, one from California that can give rise to fertile offspring up to 28°C and one from Japan that is fertile up to 30°C. We show that the optimum temperature and the upper temperature limit for fertility is shifted higher in the Japanese strain suggesting that there is a mechanism that controls the temperature response of fertility across a range of temperatures. By crossing the two strains, and using genetic mapping, we identify a region on chromosome V that is responsible for maintaining fertility at higher temperatures. Thus, we conclude that fitness of *P. pacificus* at high temperature is under genetic control, suggesting that it could be subject to natural selection.

## Abstract (precis)

**Summary:** Here we describe a phenotype in *Pristionchus pacificus* responsible for increased fitness at higher temperatures, and present mapping of a locus responsible for this ability in two natural isolates.

## INTRODUCTION

Most organisms live in an environment in which temperature varies over a vast range of both time and length scales: for instance with the day, with the seasons, by altitude, latitude and soil depth. Extreme temperatures are an important stress factor affecting both cellular processes and the whole organism systemically. This can lead to decreased fitness and loss of reproductive output, for example in frogs and lizards (Huey and Stevenson, 1979). Warm-blooded organisms cope with changes in ambient temperature by regulating their body temperature; however cold-blooded organisms cannot directly regulate their body temperature, and for these organisms even non-stressful temperatures affect their metabolic and developmental rates, as occurs in *Drosophila melanogaster* (Powsner, 1935; Richards, 1964).

Over the last few decades, we have come to understand that cells cope with acute heat stress by using heat shock proteins, which help protect against protein aggregation (reviewed by Richter et al., 2010). However, despite the fact that temperature is such an important variable for the fitness and survival of cold-blooded organisms, we know surprisingly little about the systemic response to higher temperatures and in particular how organisms adapt to prolonged changes in temperature. With increasing evidence of climate change (Alexander et al., 2013) it is essential to understand how cold-blooded species will adapt to changes in temperature over evolutionary timescales.

There is evidence that temperature is likely a selective factor, especially in plants [*Arabidopsis thaliana* (Hancock et al., 2011); and cultivated rice *Oryza sativa ssp. japonica* (Ma et al., 2015)]. It is also known that there are different temperature preferences between and within different species of nematodes, for example *Caenorhabditis briggsae* strains that belong to the tropical clade have a larger brood size when raised at higher temperatures compared to strains from the temperate clade, which are more fertile at lower temperatures (Prasad et al., 2011). In contrast, the sister species *Caenorhabditis elegans* seems to be more prevalent in temperate climates than it is in the tropics (Prasad et al., 2011). There is also a difference in the temporal distribution of *C. elegans* and *C. briggsae*. A field study in an orchard in France has shown that populations of *C. briggsae* flourish in warmer months while *C. elegans* is more numerous in colder months because of the preferred temperatures of these two nematodes (Félix and Duveau, 2012).

The nematode *P. pacificus* is used as a model system for evolutionary biology (Sommer and McGaughran, 2013). It has a necromenic relationship with beetles including scarab beetles (Mayer et al., 2009). On the living beetle, these nematodes can be exclusively found as dauer larvae in a dormant state until the death of the host, at which time they re-enter the reproductive cycle and feed on the bacteria that proliferate on the decomposing body of the beetle (Herrmann et al., 2006; Ragsdale, 2015). This lifestyle characteristic has been exploited to build a large collection of natural isolates because nematodes can be extracted from beetles that are easily collected at light traps (Herrmann et al., 2006), a process which has been carried out at locations all over the world, including countries with different climates.

In this paper, we mine the collection of *P. pacificus* natural isolates to identify two strains with different temperature preferences. We show that a strain from Japan can give rise to fertile offspring at 30°C, 2°C higher than the archetypal strain of *P. pacificus*, which was isolated in California. Precise quantification of lifetime fecundity for both of these strains across a range of temperatures shows that the increased fitness of the Japanese strain at higher temperatures comes at the cost of decreased fitness at lower temperatures where the strain from California does better. Mapping data shows that a region of chromosome V is linked to increased fitness at high temperatures. Also, phylogenetic analysis indicates that this ability has arisen recently and independently in different lineages of *P. pacificus*.

## RESULTS AND DISCUSSION

### Effect of temperature on the archetypal *P. pacificus* strain PS312

**Fig. 1.**
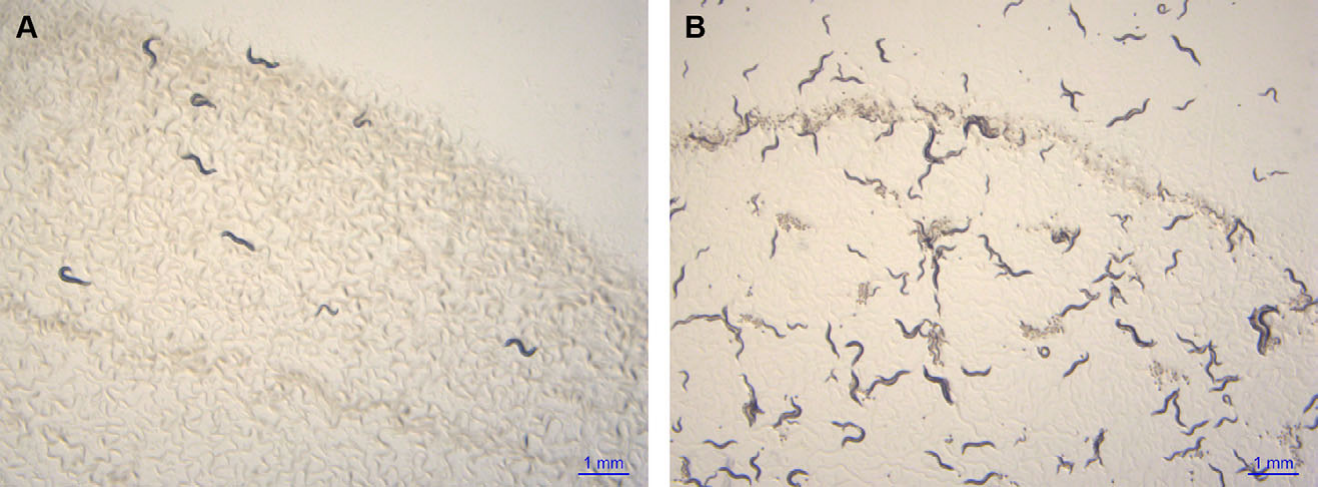
**A natural isolate of *P. pacificus* from Japan (RS5194) can give rise to fertile offspring at 30°C while a strain from California (PS312) cannot.** Individual J3 larva from strains PS312 (A) RS5194 (B) raised at 20°C were transferred to a fresh NGM plate that was incubated at 30°C for five days. Scale bar=1 mm.

We began our study by investigating the effect of temperature on the archetypal *P. pacificus* strain isolated in California called PS312 (Sommer and Sternberg, 1996). Single hermaphrodites of this strain, raised at 20°C to the third larval stage (J3), were shifted to 25°C. After 5 days they had given rise to offspring that were themselves fertile and had produced many healthy animals at mixed stages of development. However, when this experiment was repeated shifting to 30°C the founder hermaphrodite gave rise to offspring that either arrested at the larval stage or developed to adults but were sterile (Fig. 1A). By shifting animals raised at 20°C to higher temperatures in one degree increments we precisely determined the upper temperature for giving fertile offspring to be 28°C (Table 1).

**Table 1.**
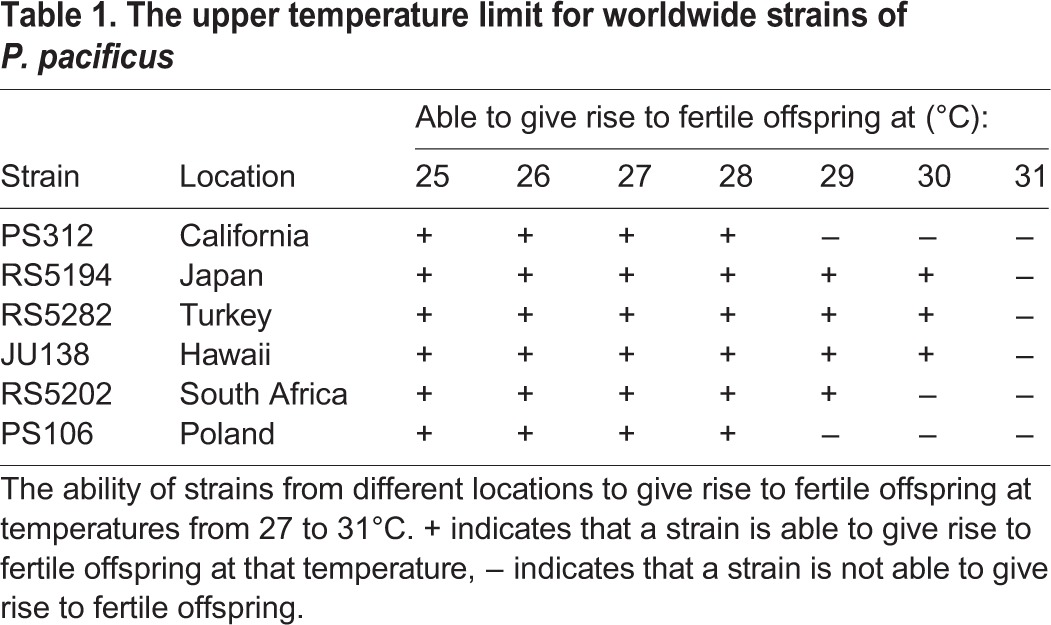
**The upper temperature limit for worldwide strains of *P. pacificus***

### *P. pacificus* strain from Japan with an increase in the upper temperature limit

Next, we tested the variability in the upper temperature limit of *P. pacificus* for natural isolates by screening a group of strains collected from countries with different climates. Differences were found, with strains having upper temperature limits for giving rise to fertile offspring between 28 and 30°C (Table 1). A strain from Japan called RS5194 was chosen for further study because it had one of the highest upper temperature limits being able to survive 2°C higher than PS312. Also, it could be propagated for many generations at 30°C and had a larger brood-size at that temperature (Fig. 1B and see below).

### Difference between two strains in their fitness across a temperature range

**Fig. 2.**
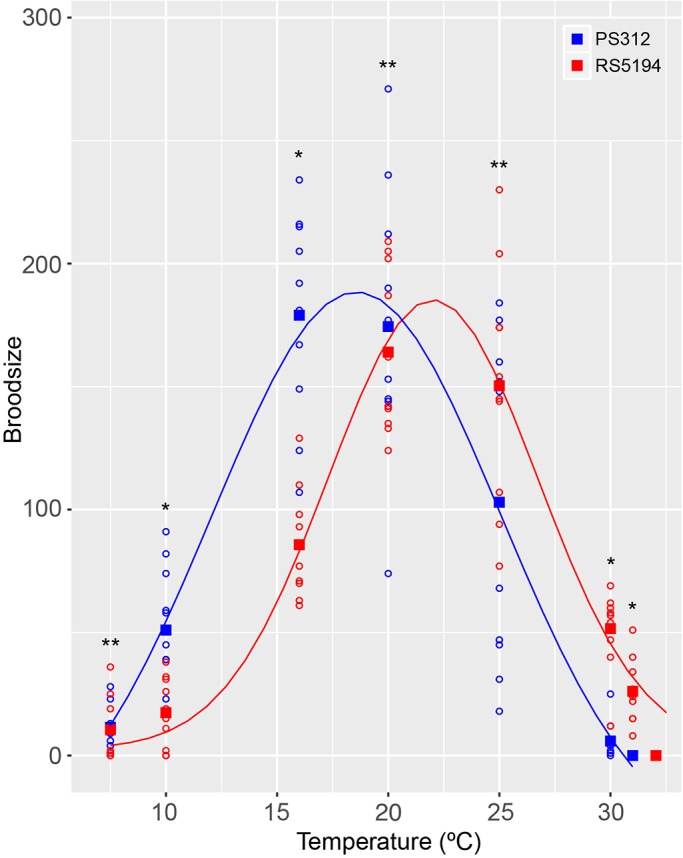
**Life time fecundity of *P. pacificus* strains PS312 (blue) and RS5194 (red).** Total number of offspring for 10 replicates, (open circles) with the mean (squares). A normal distribution is fitted to the data (solid lines). Unpaired Student's *t*-test was performed to test if there was a significant difference between the brood size of PS312 and RS5194 for each temperature. The *P*-values for each temperature are: 31°C *P*<0.0001; 30°C *P*<0.0001; 25°C *P*=0.085; 20°C *P*=0.62; 16°C *P*<0.0001; 10°C *P*=0.0031; 7.5°C *P*=0.84, * significant difference between the two strains, ** not significantly different.

To better quantify the effect of temperature on fitness across a range of temperatures, the lifetime fecundity of individual hermaphrodites of the Californian (PS312) and Japanese strains (RS5194) were quantified between 7.5 and 32°C. For strain PS312, the temperature at which the fertility was at its maximum was between 16 and 20°C (179 and 174 offspring per hermaphrodite respectively). A drop-off in fitness was observed at higher temperatures and each hermaphrodite gave on average only six offspring at 30°C and by 31°C they were totally sterile (Fig. 2). A similar decrease in fitness was observed at lower temperatures where each hermaphrodite gave on average only 11 offspring at 7.5°C. Fitting a normal distribution to the data gave an optimum temperature for fecundity of 18.8°C.

For strain RS5194, the temperature at which fertility was at its maximum was between 20 and 25°C (164 and 150 offspring per hermaphrodite respectively) and, as for PS312, there was a drop-off in fitness at temperature extremes. The estimated optimum temperature for fecundity for RS5194 was 22.2°C, which was an increase of 3.4°C compared to PS312. Interestingly, there was a gain of fitness at 30°C for RS5194, with an average of 52 offspring per hermaphrodite compared to six for PS312 (significantly different: Student's *t*-test, *P*>0.0001), but this comes at the cost of a decreased fitness at 10°C, RS5194 giving an average of 17 offspring per hermaphrodite compared to 51 for PS312 (significantly different: Student's *t*-test, *P*>0.0001). This shows that strain RS5194 has not just increased its fitness at high temperatures but has shifted its whole fitness profile higher as shown for *C. elegans* and *C. briggsae* (Begasse et al., 2015) and for populations of *D. melanogaster* from the tropical and temperate climates (Trotta et al., 2006). Therefore, two phenotypes can be defined for these strains: *P. pacificus* strain PS312 from California is low temperature tolerant (Ltt); and *P. pacificus* strain RS5194 from Japan is high temperature tolerant (Htt).

### The difference in upper temperature limit between PS312 and RS5194 has a genetic basis

To test if the difference between the upper temperature limit of *P. pacficus* strains PS312 and RS5194 had a genetic basis, they were crossed and the inheritance of the trait was followed. Initial crosses indicated that the Htt trait was dominant. To confirm this, we performed a cross with a strain of *P. pacificus* PS312 that bears a recessive *pdl-1 (sy304)* allele. Animals that have the *pdl-1* mutation have a dumpy phenotype (similar to that of *C. elegans*), having a short body morphology that can be identified using a dissecting microscope (Kenning et al., 2004; Page and Johnstone, 2007). This strain allowed us to distinguish between offspring from the cross, which should have a non-dumpy phenotype (and be heterozygous: *dpy-1(sy304)*/+), and rare offspring resulting from self-fertilisation of the hermaphrodite, which should be dumpy. If Htt is dominant, then all F1 that are non-dumpy should be able to give fertile offspring at 30°C, and the F1 that are dumpy should not be able to give fertile offspring at 30°C. For one such cross, we could obtain 238 F1 offspring, 209 were non-dumpy and 25 were dumpy (Table 2). Four animals crawled off the plate or were killed by the worm pick and were excluded (unscorable). F1 were singled then shifted to 30°C and tested for their ability to survive at 30°C. We could show that all (209/209) non-dumpy individuals were Htt and all (25/25) dumpy individuals were Ltt (Table 2). Therefore, we can conclude that Htt is dominant and 100% penetrant.

**Table 2.**
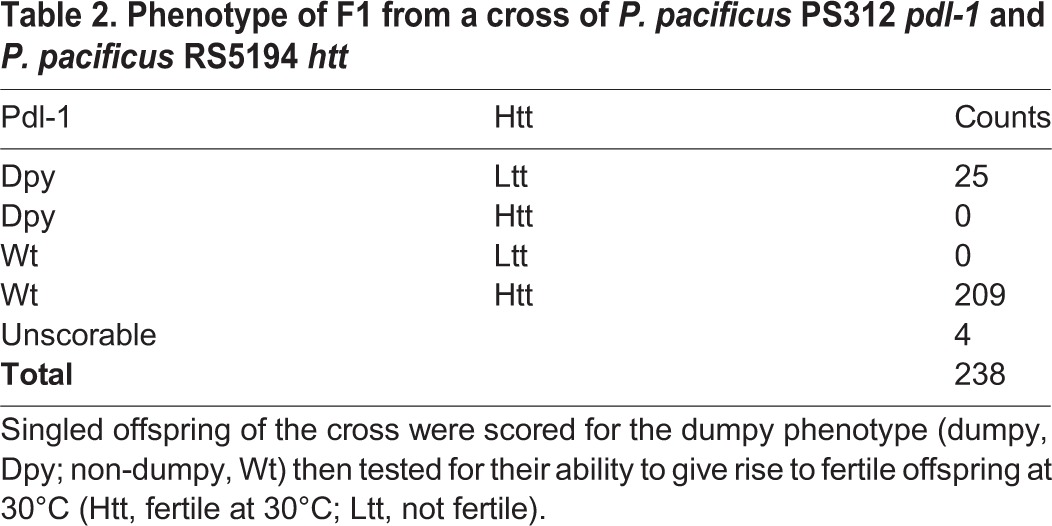
**Phenotype of F1 from a cross of *P. pacificus* PS312 *pdl-1* and *P. pacificus* RS5194 *htt***

**Fig. 3.**
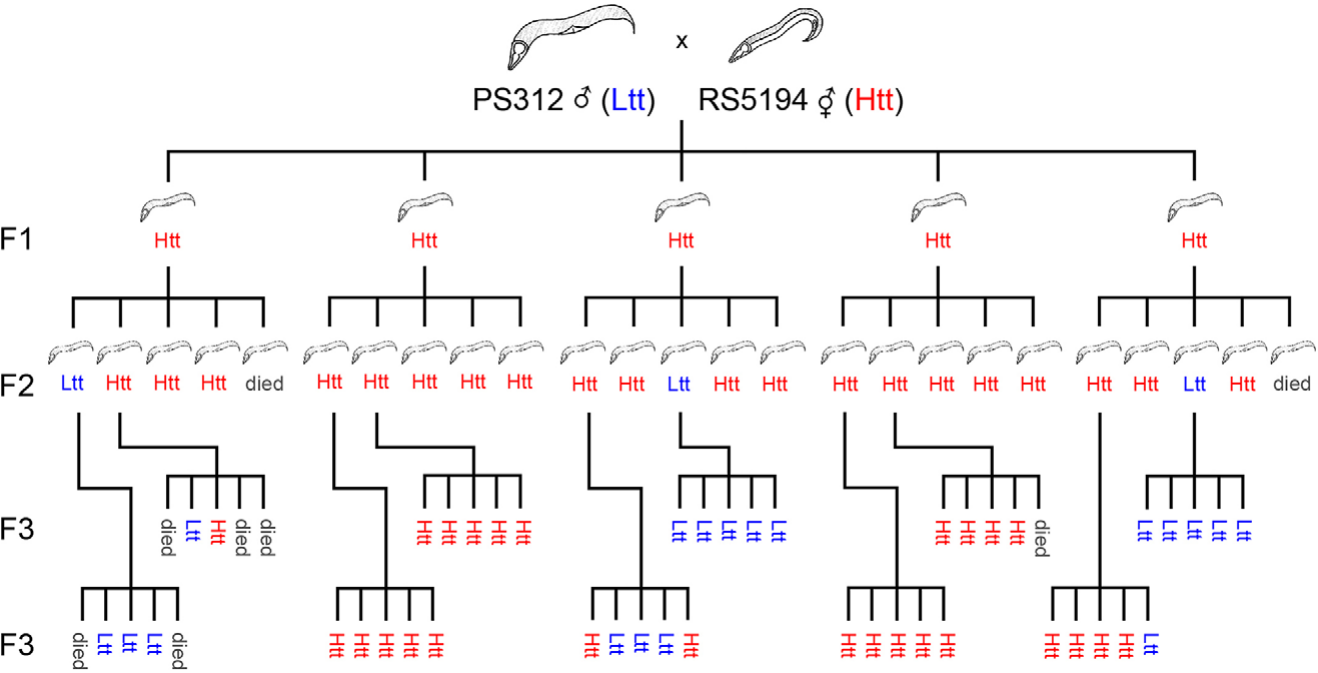
**A cross of *P. pacificus* strains PS312 and RS5194, and the phenotype of their offspring for three generations.** Males of strain RS5194, which is high temperature tolerant (Htt), were crossed with hermaphrodites of strain PS312, which is low temperature tolerant (Ltt). The phenotype of the offspring are indicated (Htt or Ltt) and animals that died before their phenotype could be scored are also shown (see Materials and methods for details).

Further crosses were performed with the wild-type strain of PS312 to prevent any effects caused by loci linked to the *pdl-1* allele. Five independent crosses were performed with hermaphrodites of the wild-type strain PS312 and males of the Htt strain RS5194, and the phenotype of their offspring followed for three generations (see Materials and methods for details). The outcome of one such cross is shown schematically in Fig. 3. In total for all crosses, 23 F1 hybrids were Htt and therefore were progeny of the cross (see above). These animals were allowed to self fertilise. The phenotype segregated in the F2 such that 82% of individuals were Htt (93/113, see Table 3). For a single dominant locus, one would expect 75% of F2 to have the phenotype, therefore the percentage of strains that we observe is higher than expected (binomial test: *P*=0.042, see Materials and methods) suggesting that there might be additional influencing loci. To investigate these possibilities further, we selected Htt and Ltt F2 individuals and tested if the trait bred true, expecting that if there were an additional recessive locus it would exhibit a phenotype in the third filial generation. 46 F2 individuals (either Htt or Ltt) were selected and five of their offspring were tested for fertility at 30°C and the genotype of the mother was inferred. All Ltt F2 that were tested gave rise only to Ltt offspring in the F3 (62 offspring from 15 F2, Table 3). From this we can infer that all Ltt F2 were homozygous *ltt* offering no evidence for a second recessive locus. From these experiments we can conclude that the simplest explanation is that Htt is controlled by a single locus. We designate such a locus as *htt* for high temperature tolerant.

**Table 3.**
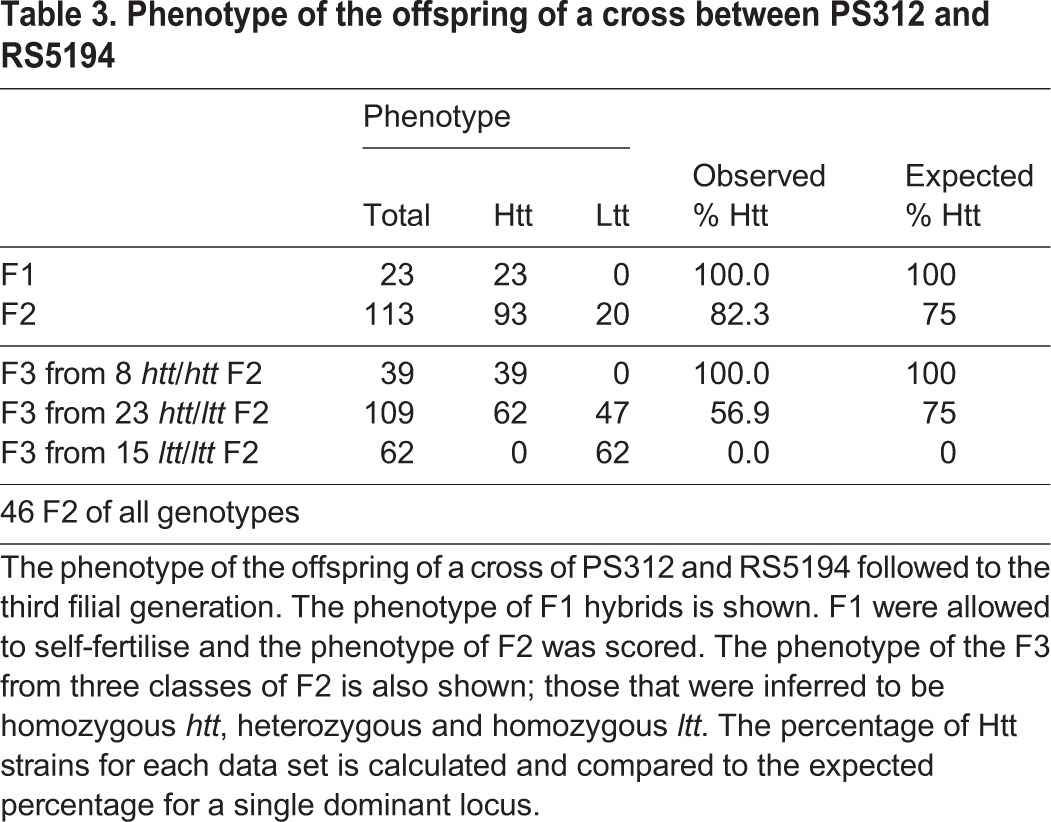
**Phenotype of the offspring of a cross between PS312 and RS5194**

### Mapping the *htt* locus

**Fig. 4.**
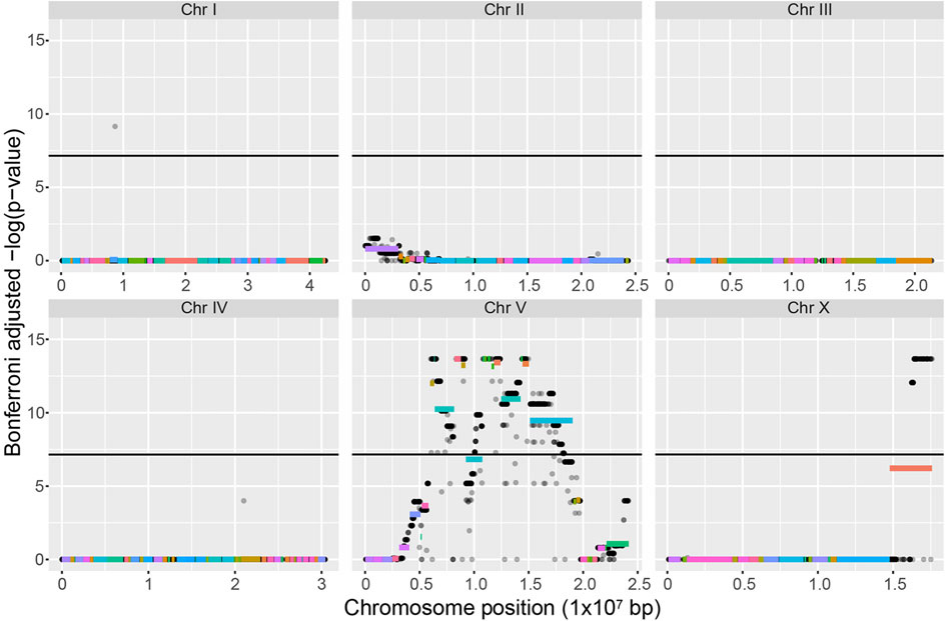
**Mapping data to locate the *htt* locus.** The statistical association between phenotype and genotype for all members of the mapping panel. Every gray point represents a variant base between PS312 and RS5194; points are transparent so appear black when overlaid. Its position on the x-axis is its position on the chromosome, and its position on the y-axis is a measure of the statistical significance of the phenotype-genotype relationship in all members of the mapping panel namely the Bonferroni adjusted −log(*P*-value). 0 means low significance, high values mean that region of the chromosome is likely to be linked to the causal mutation. The black line indicates the genome wide level of significance. The position of the contigs of the draft genome assembly are also shown as colored lines, and their position on the y-axis is the mean −log(*P*-value) for all SNPs on that contig.

If the phenotype was indeed determined by a single locus we should be able to locate it using mapping techniques. To do this, we made a mapping panel consisting of many independently derived hybrids of PR312 and RS5194. Hybrids were then inbred for 10 generations at 20°C to ensure that almost all loci are homozygous. This produced a mapping panel of 94 recombinant inbred lines (RILs) that were scored for the Htt phenotype. The genome of each of RILs was sequenced. At the chromosomal position that bears the *htt* locus, there should be a strong correlation between RILs that have the Htt phenotype and the genotype of strain RS5194. At loci that are not linked to *htt*, there should be an equal chance of inheriting the PS312 or the RS5194 genotype. Fig. 4 summarises the mapping data where the strong correlation between genotype and phenotype is indicated by a high significance level [−log(*P*-value), see Materials and methods for details]. This identified the central part of chromosome V and the right flank of chromosome X that falls above the significance threshold for association with the phenotype. Within these two regions are 12 large contigs consisting of 5.23 Mb of sequence that have the highest level of significance which represent the most likely position for the causative mutation (Fig. S1). We would expect a smoother increase in the signal on either side of this region. However, we see discontinuities that are likely due to incorrect assembly of the reference genome or chromosome rearrangements in the strain RS5194, especially the right flank of contig10 on chromosome X. These contigs likely map to one continuous region on chromosome V.

Further mapping experiments are ongoing with the aim to reduce the size of the region of interest and precisely identify the causative mutation, as the current region of interest is too large for a candidate approach. We have performed one preliminary experiment on a likely candidate in this region, contig22-snap.11 that encodes a protein similar to a small heat shock protein, HSP27 (Garrido et al., 2012). However, over expression of contig22-snap.11 was not sufficient to convey the ability to survive at 30°C in the PS312 background (see Materials and methods for details).

### Evolution of the Htt trait

**Fig. 5.**
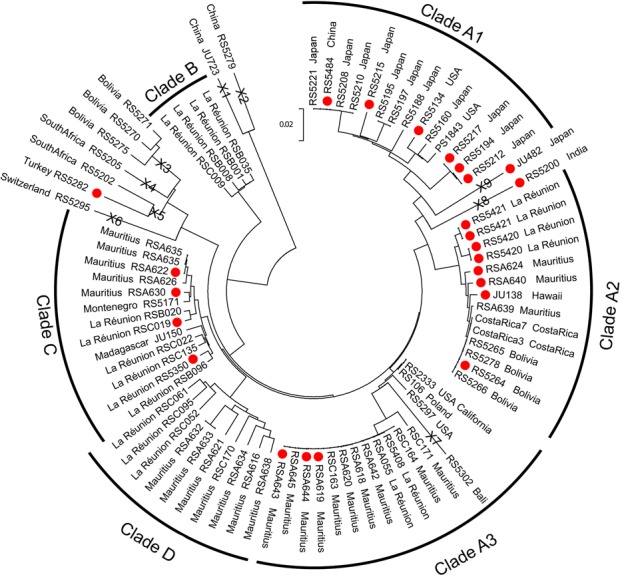
**A phylogenetic tree of strains of *P. pacificus* collected from worldwide locations.** Strains that are able to give fertile offspring at 30°C are shown with red circles. The country where the strains were collected from is also indicated. The name of each clade is indicated and divergent outliers are indicated with the designation X1-X9.

To see how the ability to survive at 30°C has arisen, we constructed a phylogenetic tree of strains of *P. pacificus* collected from worldwide locations (Fig. 5). As previously shown, worldwide strains of *P. pacificus* fall into many clades (Morgan et al., 2011); we designate these clades A1, A2, A3, B, C and D (Fig. 5, and see Materials and methods). There are several isolates that are relatively divergent and fall outside of these clades (designated X1-X9), for example strains RS5279 and JU723 (Fig. 5), which effectively root the tree (Baskaran and Rödelsperger, 2015). Htt strains are found in clades A1-A3, clades C and X5. This indicates that the trait has arisen multiple times independently. Although we cannot rule out that this phenotype has spread between individuals in different clades by outcrossing, this seems unlikely to have occurred between strains found in different countries or even different continents. The ancestor of all *P.* *pacificus* was likely to be Ltt as the majority of strains are Ltt and all strains in the deep-rooted clade B and outliers JU723 and RS5279 are Ltt. This suggests that the ability to survive at 30°C has arisen recently and is a derived trait.

## CONCLUSIONS

We present two lines of evidence that support the conclusion that the ability to give rise to fertile offspring at 30°C is controlled by a single locus. Firstly, the cross of PS312 and RS5194 suggests that the phenotype is controlled by a single locus segregating according to Mendelian laws; however, more complicated scenarios are possible, for example multiple loci with small effect sizes that affect fitness could influence the phenotype. The second line of evidence is that the mapping data showed that the phenotype is associated with a single region of chromosome V.

The locus that we identify might represent a single nucleotide polymorphism in a gene or many SNPs in one or more genes that influence the phenotype. Further experiments are required to narrow down the region of interest and identify the causative SNP(s). However, certain qualities of the causative mutation can be inferred from the data we present. Given that Htt is dominant, the phenotype should be due to a gain-of-function mutation in strain RS5194. Broadly speaking, this means that the causative SNP(s) convey additional function to a gene product that somehow affects fitness in such a way that it functions better at higher temperatures. Alternatively, a dominant negative mutation in a negative regulator would behave similarly. Conversely, Ltt could be considered to be recessive and the causative mutation could be a loss-of-function mutation, meaning that it results in a decrease in activity at higher temperatures and corresponding loss of fitness. We find the former explanation to be more likely, because a loss-of-function mutation in the Ltt strain would not explain the increase of fitness that we observe at lower temperatures for PS312. Because it seems likely that the last common ancestor of all *P. pacificus* was low temperature tolerant we infer that the ability to give fertile offspring at 30°C is a recently derived trait and therefore a gain-of-function mutation.

We do not know the molecular identity of the locus we identify, but it seems likely that the causative mutation is a gene that, in some way, affects the robustness of the gonad in response to temperature. The key to understanding this might come from a better understanding of how sperm is affected by temperature in hermaphrodites (Harvey and Viney, 2007). It is well known that the early development of an organism is particularly sensitive to temperature, for example bees maintain the temperature of their hive at the optimum temperature for the development of their larva. However, there is still relatively little known about what causes this sensitivity. Begasse et al. (2015) showed that at high temperature there were cell cycle defects during early embryogenesis in *C. elegans* and *C. briggsae*. Furthermore, there was a breakdown in the temperature dependence of the rate of the cell division at temperatures close to the upper temperature limit for these species. It was suggested that these phenomena are, in part, defining the upper temperature limit of fertility in these two model nematodes. Further work in the two *P. pacificus* strains we describe here will shed light on this phenomenon.

In conclusion, we have identified a 5.23 Mb locus in *P. pacificus* that confers high temperature tolerance in strain RS5194. Further work is required to identify the causative mutation and determine its mode of action. Extending the number of strains studied in future work could identify more interesting strains with different temperature-related phenotypes. This raises the exciting possibility that the independent acquisitions of this phenotype represent convergent evolution of the trait through independent gain-of-function mutations in a conserved pathway that regulates the thermal response of fitness. Replicating the mapping approach described in this paper with other high temperature tolerant strains from other clades might identify different genes in such a pathway. Also, further work is required on the Htt trait in natural populations of nematodes to test if this phenotype is advantageous when nematodes are subjected to high temperatures in their natural habitat, specifically to test if it is subject to natural selection.

## MATERIALS AND METHODS

### Strain maintenance

*P. pacificus* was propagated according to standard methods for *C. elegans* (Brenner, 1974). Briefly, lines were propagated on NGM plates inoculated with *Escherichia coli* OP50. In order to minimise epigenetic effects, all strains were propagated at 20°C for at least two generations on abundant food before temperature experiments were performed. All strains are available upon request.

### Temperature shift

Carefully staged J3 larva picked from mixed stage plates grown at 20°C were singled to new NGM plates that were then shifted to either 25°C or 30°C. Strains were incubated for 5 days then their offspring were imaged on a Leica M205 video dissecting microscope.

### Lifetime fecundity

To measure the average number of offspring per hermaphrodite, 10 J3 larva from mixed stage plates grown at 20°C were singled onto NGM agar plates inoculated with OP50 and shifted to the test temperature. Temperatures used were 7.5, 10, 16, 20, 25, 30 and 31°C. To prevent plates from overcrowding and to ensure that only the F1 generation was counted, founder hermaphrodites were picked on to fresh plates each day. After a further 3 days incubation, the number of hatched larva were counted on each plate and the number of offspring per individual hermaphrodite was calculated. From these numbers, the mean number of offspring per animal was calculated for strains PS312 and RS5194. Plots show the mean (squares) and individual values for each of the 10 repeats (empty circles) (Fig. 2).

### Cross PS312 pdl-1×RS5194

Three males of strain RS5194 and one hermaphrodite of strain PS312 *pdl-1* (*sy304*) were placed on a plate seeded with *E. coli* OP50. Several such plates were incubated at 20°C until the F1 had developed to the J2 or J3 larva stage. Well-mated hermaphrodites were identified by the presence of males in the first filial generation. Hybrid J3 hermaphrodites from these plates were singled to fresh plates and shifted to 30°C and incubated for 7 days. The plates were then inspected and scored for the dumpy phenotype and their ability to give rise to fertile offspring.

### Cross PS312×RS5194

Crosses with hermaphrodites of the wild-type strain PS312 and males of strain RS5194 were performed and the phenotype of their offspring followed for three generations. As Ltt strains do not survive at 30°C, they could not be propagated past the F1 using the protocol described above. Instead, F1 hermaphrodites were cultivated at 20°C and individual hermaphrodites were singled for every generation. We then took five of their offspring and assayed the phenotype by shifting them to 30°C and used that data to infer the phenotype of the mother. As we assumed Htt to be dominant, three out of four of the offspring of a homozygous individual should be also have been Htt, therefore if any of the five offspring tested were giving fertile offspring at 30°C then the mother was designated as Htt. For a typical cross, one hermaphrodite was placed on an NGM plate inoculated with a small volume of OP50 together with three males. Five offspring from well-mated hermaphrodites were singled and incubation continued at 20°C. Once the F1 had laid eggs, the mothers were picked onto fresh plates and stored until their phenotype could be inferred. This was repeated for each generation for a total of 25 F2 and 50 F3. In later repeats we did not select randomly which F2 to test, instead we chose more Ltt to specifically test the hypothesis that a recessive trait might segregate a phenotype in the F3. Occasionally, worms were killed during transfer or crawled off the plate and dried out before they could be properly scored for heat tolerance, resulting in a reduced total number of individual animals tested. To account for this and increase the number of repeats, such a cross was repeated five times independently. Excluding worms for which there was not a complete pedigree did not affect the outcome of the experiment. Each repeat gave similar results and one typical experiment is shown graphically in Fig. 3. A binomial test was used to distinguish between the percentage of Htt strains expected in the second generation and that expected by a single dominant locus. The quoted *P*-value is the probability of observing 93 Htt strains or more out of 113 trials assuming the null hypothesis that the probability of observing the Htt phenotype is 0.75.

### Mapping

Hybrids were made by mating one hermaphrodite of PS312 with three males of RS5194. Heterozygous F1 individuals were identified by selection at 30°C. 400 single F2 animals were transferred to new plates and allowed to self-fertilise at 20°C. For each F2, one single offspring larva was randomly chosen and transferred to a new plate for every generation until the F10 for each of the 400 independent lines was reached, all the time being cultivated at 20°C. 214 lines survived the inbreeding. The 186 strains lost were either killed during the transfer process, crawled onto the wall of the petri dish and dried out, or because of genetic incompatibilities between the two parental strains. The number of strains lost is consistent with previous mapping panels made for *P. pacificus* (Kienle and Sommer, 2013). The phenotype of each surviving recombinant inbred line (RIL) was determined: 89 were Htt and 125 were Ltt, suggesting that there is some underestimation of the number of Htt strains possibly due to outbreeding suppression that would reduce the fitness of hybrids across all temperatures. The genome was sequenced of 94 strains representing equal numbers of Htt and Ltt individuals with the most clear-cut phenotypes. The two parental strains were also re-sequenced as controls, giving 96 strains in total. SNPs between the two parental lines were detected and the genotype at every variant position was identified in each of the RILs. Only SNPs covered by 25 or more reads and with a quality score of 10 or more were included in the final analysis. A *P*-value for the correlation between the genotype at every variant position and the phenotype for each of the RILs was calculated and plotted on a SNP by SNP basis (Fig. 4). The *P*-values were adjusted using the Bonferroni correction method (Miller, 1981; Benjamini and Hochberg, 1995; Qu et al., 2010), as implemented in the *p.adjust* function in R. The significance threshold for the adjusted *P*-value was set to 0.001/Total # of SNPs (α=0.001/14,173=7.05×10^−8^) (Duggal et al., 2008). The two lone SNPs with high −log(*P*-value), one each on chromosome I and chromosome IV, and the right flank of contig10 on chromosome X probably represent misaligned sequences in the draft genome of *P. pacificus*. Most likely these regions would be found somewhere in chromosome V in a future, improved assembly of the genome.

### Construction of strains

A strain overexpressing contig22-snap.11, which is predicted to encode a homolog of Hsp-27, and 5 KB of the promoter region and 2 KB of the downstream region was made by injection. *rfp* fused to a constitutive promoter was co-injected and acted as a marker to screen for positive lines (see Schlager et al., 2009 for details). Three independent lines were tested and all showed the same phenotype.

### Construction of the phylogenetic tree

Variant bases were identified in a custom pipeline implemented in Perl and Unix (Christiansen et al., 2012; Keringhan and Pike, 1983). Briefly, whole genome sequences were obtained and variant bases were identified. The genotype of all strains at all variant positions was extracted from the genome sequence covered by the largest 49 contigs. 10,000 SNPs were selected and concatenated. This sequence was used to construct a neighbor joining tree in Mega 6 (Tamura et al., 2013). Clade names are based on the previous mitochondrial clade assignments of Herrmann et al., (2010) and refined based on whole genome data (Rödelsperger et al., 2014) (Fig. 5). Data are available upon request.
